# A Consensus Approach to the Incorporation of Total Neoadjuvant Therapy in a Treatment Algorithm for Stage I–III Resectable Rectal Cancer

**DOI:** 10.3390/curroncol33050274

**Published:** 2026-05-08

**Authors:** Sami A. Chadi, Karineh Kazazian, Paul Savage, Christine Brezden-Masley, Ron Burkes, Eric Chen, Anand Govindarajan, Ali Hosni, Raymond Jang, Erin Kennedy, John Kim, Jelena Lukovic, Aruz Mesci, Catherine O’Brien, Fayez Quereshy, Abdulazeez Salawu, Peter K. Stotland, Carol J. Swallow

**Affiliations:** 1 Sprott Department of Surgery, Division of General Surgery and Surgical Oncology, University Health Network and Princess Margaret Cancer Centre, Toronto, ON M5G 2C4, Canada; karineh.kazazian2@uhn.ca (K.K.); fayez.quereshy@uhn.ca (F.Q.); 2 Department of Surgery, University of Toronto, Toronto, ON M5T 1P5, Canada; paul.savage@mail.utoronto.ca (P.S.); anand.govindarajan@sinaihealth.ca (A.G.); erin.kennedy@sinaihealth.ca (E.K.); peter.stotland@nygh.on.ca (P.K.S.); carol.swallow@sinaihealth.ca (C.J.S.); 3 Department of Medicine, Mount Sinai Hospital, Sinai Health, Toronto, ON M5G 1X5, Canada; christine.brezden@sinaihealth.ca (C.B.-M.); ron.burkes@sinaihealth.ca (R.B.); abdulazeez.salawu@uhn.ca (A.S.); 4 Department of Medicine, Temerty Faculty of Medicine, University of Toronto, Toronto, ON M5S 3H2, Canada; eric.chen@uhn.ca (E.C.); raymond.jang@uhn.ca (R.J.); 5 Department of Medical Oncology, Princess Margaret Cancer Centre, University Health Network, Toronto, ON M5G 2C4, Canada; 6 Department of Surgery, Mount Sinai Hospital, Sinai Health, Toronto, ON M5G 1X5, Canada; 7 Department of Radiation Oncology, Princess Margaret Cancer Centre, University Health Network, Toronto, ON M5G 2M9, Canada; ali.hosni@uhn.ca (A.H.); john.kim@uhn.ca (J.K.); jelena.lukovic@uhn.ca (J.L.); aruz.mesci@uhn.ca (A.M.); 8 Department of Radiation Oncology, University of Toronto, Toronto, ON M5T 1P5, Canada; 9 Department of Surgery, North York General Hospital, Toronto, ON M2K 1E1, Canada

**Keywords:** total neoadjuvant therapy, rectal cancer, non-operative management, treatment algorithm, consensus

## Abstract

Advances in surgical techniques, radiographic imaging capabilities, radiotherapy, and chemotherapy have led to improved outcomes for patients with rectal adenocarcinoma. Treatment strategies have correspondingly evolved, as seen with total neoadjuvant therapy (TNT) and organ preservation approaches. TNT is a treatment strategy for primary, non-metastatic, resectable mismatch repair proficient rectal cancer where the intent is to administer all appropriate adjuvant therapy in the preoperative phase, including both systemic therapy and chemoradiotherapy/radiotherapy. In this setting, TNT is increasingly administered for the purposes of maximizing tumour response to facilitate resection, improving treatment compliance, thus increasing the likelihood of a complete response to allow for organ preservation and for the possibility of improving survival. While several recent randomized controlled trials have described the role of TNT in the contemporary treatment of rectal cancer, there is significant heterogeneity in sequencing of treatments, dosing, allowance for non-operative management, and the potential for over-treatment. Our objective here was to incorporate current evidence to develop a consensus-based institutional treatment algorithm to be used in the ambulatory and multidisciplinary team setting for the treatment of stage I–III rectal cancer.

## Simple Summary

For nearly two decades, the management paradigm for patients with rectal cancer has been ppreoperativeradiation and surgery followed by chemotherapy. This has evolved significantly in recent years with the advent of total neoadjuvant therapy (TNT) and non-operative management. In TNT, patients receive radiation and chemotherapy in the pre-operative phase with the goal of facilitating resection, improving treatment compliance, and increasing the likelihood of a complete response, which may allow for some patients to avoid surgery, known as non-operative management. While several recent clinical trials support TNT, they vary with respect to patient inclusion criteria, treatment sequencing, and allowance for non-operative management, which has led to significant heterogeneity in the implementation of these strategies. To overcome this, the current study was designed to summarize existing evidence and develop a practical, consensus-based treatment algorithm that incorporates tumour-specific risk features while balancing treatment efficacy and toxicity.

## 1. Introduction

Management strategies for rectal cancer are currently in evolution, in particular with the introduction of total neoadjuvant therapy (TNT) and non-operative management (NOM) strategies. Over the past two decades, trimodality therapy of rectal cancer with the standard of chemoradiation (CRT)/radiotherapy (RT), high-quality surgery and adjuvant chemotherapy has allowed for excellent locoregional control and a 5-year overall survival between 72% and 90%, depending on the stage of non-metastatic disease [1,2,3,4]. Dramatic improvements in response and organ preservation rates have recently been observed with neoadjuvant checkpoint blockade among the 5–10% of locally advanced rectal cancers with mismatch repair deficiency (dMMR), yet patients with MMR-proficient (pMMR) tumours continue to rely on conventional modalities [5]. Outcomes remain suboptimal, as approximately 25% of patients with locally advanced rectal cancer will experience distant recurrence within 5 years, and 40–60% of those who should receive chemotherapy after surgery either do not complete or experience delays in their intended course of systemic therapy [3,6]. Additionally, receipt of adjuvant chemotherapy significantly increases the time to ostomy reversal, with an associated impact on quality of life [7]. The concept of delivering all indicated adjuvant treatments for rectal cancer in the preoperative setting, referred to as TNT, was introduced to address these gaps in care. TNT can achieve several important short-term objectives including tumour downsizing for surgical resectability, opportunities for a clinical complete response (cCR) and organ preservation, as well as improved compliance to systemic therapy. TNT also confers the theoretical benefit of treating the tumour in its in vivo state, as well as any microscopic systemic disease earlier.

While the clinical adoption of TNT and NOM protocols in response to the emergence of new evidence has been fairly rapid and widespread, there remains a research-to-practice gap in the consolidation of the evidence to guide management decisions. We had similarly observed considerable heterogeneity in the choice of treatment strategies for non-metastatic rectal cancer at our own centre. This occurred despite the availability of treatment guidelines from organizations such as the National Comprehensive Cancer Network (NCCN) and the European Society for Medical Oncology (ESMO), which highlighted some of their limitations [8,9]. This includes a heavy reliance of the NCCN guidelines on TNM staging, with minimal incorporation of MRI-defined risk factors (e.g., distinction between T3a/b versus cT3c/d, mesorectal fascia involvement, extramural venous invasion, N2 or lateral pelvic lymph node involvement) and tumour height, which is a major determinant of locoregional and distant recurrence risk, neoadjuvant chemoradiation efficacy, and potential for sphincter-sparing surgery and postoperative bowel dysfunction. While tumour height and MRI-defined risk factors are incorporated into the ESMO guidelines, there are several branchpoints with multiple potential treatment options with little to no explanation to guide decision-making.

With the aim of closing this gap, we took a rigorous, stepwise approach to creating an evidence-based consensus within the gastrointestinal division of our cancer centre, including a full-day expert panel discussion with representation from all disciplines. With the help of extensive validation exercises, we reached consensus on a practical algorithm to guide the treatment of cases of non-metastatic resectable rectal adenocarcinoma at our centre, that was also adapted for use at other neighbouring centres in our province. Based on this process, we present the rationale and strategy we took in developing this evidence-based approach for incorporating TNT into the overall rectal cancer treatment algorithm.

## 2. The Evidence

### 2.1. Total Neoadjuvant Therapy

Retrospective and multicentred phase II studies initially showed the feasibility of TNT, with relatively high rates of pathologic complete response (pCR; 22.4–36%) [10,11,12,13]. Prolongation of the interval between completion of long-course CRT and surgery by progressively longer periods of consolidation chemotherapy was associated with increased response rates: pCR rates of 18% with CRT alone, 25% with two cycles of consolidation mFOLFOX6, 30% with four cycles, and 38% with six cycles were observed by Garcia-Aguilar and colleagues [11]. The effectiveness of select TNT protocols was then examined in two large randomized control trials: PRODIGE23 (Partenariat de Recherche en Oncologie Digestive) and RAPIDO (Rectal Cancer and Preoperative Induction Therapy Followed by Dedicated Operation) (Table 1) [14,15]. Each of these multicentre phase III trials compared morbidity and outcomes in patients with locally advanced rectal cancer treated with TNT to those in patients treated with the conventional approach of preoperative CRT and postoperative chemotherapy.

PRODIGE 23 randomized 461 patients with clinical T3 or T4 rectal cancer to either preoperative CRT, total mesorectal excision (TME) surgery and postoperative FOLFOX/CAPOX (conventional approach), or preoperative induction with mFOLFIRINOX followed by CRT, TME surgery, and postoperative FOLFOX/CAPOX (TNT approach) [14]. The group of patients randomized to TNT showed high treatment compliance (92%), a higher rate of pCR (27.8% vs. 12.1%; *p* = 0.002), improved disease-free survival (DFS; 76% vs. 69%; *p* = 0.034) and reduced distant metastases (17% vs. 25%) at 3 years, with no impact on surgical morbidity. After long-term follow-up, 7-year overall survival was significantly longer in patients who received TNT (81.9% vs. 76.1%; *p* = 0.033) [16]. While the inclusion of neoadjuvant chemotherapy in the TNT arm resulted in improvement of tumour-related symptoms, there were also transient decreases across most health-related quality of life functional scores [17].

RAPIDO randomized 920 patients to either long-course CRT, TME surgery, and postoperative FOLFOX/CAPOX (standard approach) or short-course RT followed by FOLFOX/CAPOX and then TME surgery (TNT approach) [15]. Included patients had high-risk primary tumours with at least one MRI-defined risk factor (cT4 disease, extramural venous invasion (EMVI), N2 disease, involved mesorectal fascia (MRF), or positive lateral pelvic lymph nodes). As in the PRODIGE 23 trial, treatment with TNT was associated with higher pCR rates (28.4% vs. 14.3%; *p* < 0.001) and a decrease in disease-related treatment failure (23.7% vs. 30.4%; *p* = 0.019), with similar rates of treatment-related adverse events (38% vs. 34%). Approximately 50% of patients in the standard therapy arm did not receive postoperative systemic therapy. Despite the improvements in disease-related treatment failure, no overall survival benefit was observed at 5 years (81.7% vs. 80.2%; *p* = 0.5), which has been attributed to the neoadjuvant Therapy-Related Shortening of Survival (ATRESS) phenomenon [18]. In RAPIDO, shorter survival was observed after the onset of distant metastasis in the TNT arm (2.4 vs. 3.1 years; *p* = 0.04), which was not the case in PRODIGE 23 [16]. Another important finding from RAPIDO came with longer follow-up, where increased locoregional recurrence (10% vs. 6% at 5 years; *p* = 0.027), particularly with involvement of the MRF, was noted in the TNT arm, raising concerns regarding the inadequacy of using short-course RT in this setting [19]. Health-related quality of life and low anterior resection syndrome scores were similar between groups up to 36 months, and neurologic toxicity was similar among patients receiving oxaliplatin whether in the pre- or postoperative setting [20].

As in RAPIDO, the STELLAR (Short-Term Radiotherapy Plus Chemotherapy Versus Long-Term Chemoradiotherapy in Locally Advanced Rectal Cancer) phase III randomized trial utilized short-course RT in a TNT approach. STELLAR randomized 599 patients with cT3/T4 or node-positive rectal cancer to either preoperative short-course RT followed by four cycles of CAPOX or conventional CRT, followed by TME surgery and additional chemotherapy (two cycles of CAPOX for the short-course group, and six cycles for the standard CRT arm) [21]. At a median follow-up of 35 months, three-year DFS was equivalent in the TNT and standard treatment groups (64.5 vs. 62.3%, HR 0.883) and there were no differences in metastasis-free survival or locoregional recurrence. Interestingly, the TNT group had a higher three-year overall survival (86.5 vs. 75.1%, *p* = 0.033). While longer-term survival data are awaited, no significant differences in health-related quality of life or anal function scores were observed between the TNT and conventional CRT arms after a median follow-up of over 6 years [22].

To evaluate the optimal sequencing of chemotherapy and CRT in the preoperative phase, CAO/ARO/AIO-12 compared outcomes in patients treated with either induction FOLFOX (3 cycles) followed by CRT or CRT followed by consolidation FOLFOX (3 cycles), before TME surgery [23]. CRT followed by consolidation chemotherapy was associated with higher pCR (25% vs. 17%; *p* < 0.001) and better compliance to the intended CRT regimen, but worse compliance to chemotherapy [23]. There was no difference in DFS, locoregional recurrence or distant metastases [24]. Overall chronic toxicity (grade 3–4) did not differ between the groups, and no difference in surgical complications was noted; however, there were slightly higher rates of erectile dysfunction/impotence and vaginal dryness/dyspareunia in the consolidation chemotherapy arm [23,24,25].

### 2.2. Non-Operative Management

Sexual and/or urinary dysfunction occur in approximately 25% of patients treated by radical surgery, even with meticulous nerve-sparing techniques and in highly specialized centres [26,27,28]. The preference of patients to avoid a permanent colostomy or the dysfunction associated with a low colorectal or coloanal anastomosis, as well as the rates of pCR observed in the studies described above, raises the question of whether surgery could be safely omitted in patients with a cCR to TNT. In most high-volume centres, NOM has become an acceptable alternative for patients who achieve a cCR following neoadjuvant therapy, meaning that there is no evidence of residual tumour on digital rectal examination (DRE), rectal MRI, and direct endoscopic evaluation. TNT is considered in cases to enhance the opportunity for NOM, but only with the guidance of an experienced multidisciplinary team.

More than two decades have passed since NOM was initially proposed as a viable strategy for select patients who achieve a cCR after conventional CRT [29]. Several single-institution studies, the findings from the International Watch & Wait Database, and various meta-analyses have now confirmed favourable outcomes with NOM [30,31,32]. The OPRA trial randomized patients with stage II or III rectal cancer to either induction FOLFOX/CAPOX followed by long-course CRT or long-course CRT followed by consolidation FOLFOX/CAPOX; those with either a cCR or a near-cCR were offered observation without surgery [33]. At a median follow-up of 3 years, the primary endpoint of DFS was equivalent for the two arms (76% and 76%) and was not superior to a historical rate of 75% in patients undergoing conventional CRT followed by surgery. Distant metastasis-free survival at 3 years was also similar between the two arms (81% vs. 83%, respectively; *p* = 0.86). However, consolidation chemotherapy was associated with an increased rate of organ preservation at 5 years (54% vs. 39%; *p* = 0.012), highlighting another motivation for pursuing the TNT approach to stage II/III rectal cancer [34]. While the inclusion of patients with a near-cCR allowed for broader enrollment in the NOM pathway, a secondary analysis revealed lower rates of 3-year TME-free survival compared to patients who sustained a cCR (40% vs. 77%; *p* < 0.001) [35]. Given variability in the definitions of a near-cCR, expert-based consensus statements were created to define the clinical features and ultimately recommended this be used as a temporary entity only within 6 months of completing neoadjuvant therapy [36].

Concerns about NOM have recently been raised based on retrospective analyses demonstrating that patients with local regrowth following neoadjuvant CRT and NOM have higher rates of distant metastasis compared to patients managed by upfront TME with a near-pCR (22.8% vs. 10.2%; *p* = 0.001) [37,38]. Critics argue that the control group likely contains a heterogeneous group of patients, some of whom would have had cCR or pCR with longer intervals to response evaluation, and would thus bias the surgical cohort to favourable outcomes. Furthermore, the majority of these studies are based on populations treated with neoadjuvant CRT as opposed to TNT, and smaller series have shown comparable distant metastasis-free survival between these groups in the setting of TNT [39]. Similarly in OPRA, patients who were recommended to undergo TME after local regrowth did not display an increased rate of distant metastasis compared to those who were recommended to undergo TME following initial response evaluation [40]. Mechanistically, it remains unclear whether leaving a clinically occult residual primary tumour in situ during NOM causally results in increased metastatic spread which could theoretically be addressed by surgery, or if local regrowth reflects adverse tumour biology with inherently increased metastatic propensity.

### 2.3. Concerns Regarding Potential for Over-Treatment

As TNT protocols have proliferated and been widely implemented, there has been concurrent interest in more accurately identifying patients who will not benefit from such intensification of treatment. Several studies have sought to identify MRI features that could permit safe omission of CRT/RT. For example, the MERCURY (Magnetic Resonance Imaging and Rectal Cancer European Equivalence) and QuickSilver trials specified low-risk features on the pretreatment MRI, including distance of >1 mm to the MRF, T3 tumours greater than 5 cm from the anal verge, no evidence of lateral pelvic lymph node disease, and absent or unequivocal EMVI, that if all present led to upfront surgery with no preoperative therapy [41,42]. Patients who meet these criteria have CRM microscopic positivity rates of 2.8–7.9% and local recurrence rates of 2–7% when treated with upfront high-quality TME surgery.

Motivation to avoid over-treatment is based on the potential for harm in terms of acute and chronic treatment-related toxicities, as well as impact on quality of life. The risks of radiation-associated bowel, bladder and sexual dysfunction with decreased quality of life have been previously reported [43,44]. Clearly, systemic therapy can also result in long-term consequence, such as persistent oxaliplatin-related neuropathy. In the absence of node positivity, adjuvant chemotherapy following preoperative CRT has had limited demonstrable oncologic benefit according to several studies [45,46,47,48,49,50].

At the present time, multimodal treatment that includes both radiation and systemic therapy remains the standard of care for locally advanced rectal cancer, and it is against this standard that any new/alternative strategies must be judged. Any incremental benefit in terms of cancer control must be balanced against the risk of harm. Since radiation to the pelvis results in not only short- and long-term toxicities that include infertility, bowel, bladder, and sexual dysfunction, but also increased risk of subsequent malignancy, there is interest in determining the potential indications for safe omission of RT [43,51,52,53,54].

A salient example is the response-guided omission of RT following systemic therapy examined in the PROSPECT trial (Table 1) [55]. In this phase III trial, 1194 patients with non-metastatic clinical T2N1 or T3N0/1 rectal adenocarcinoma who were candidates for sphincter-sparing surgery were randomized to either upfront CRT (standard arm) or neoadjuvant FOLFOX and then response-guided use of CRT (experimental arm; patients with ≥20% reduction in tumour volume following neoadjuvant FOLFOX went on to surgery while those with <20% reduction in tumour volume underwent CRT followed by surgery); postoperative FOLFOX was suggested in both arms and delivered to 77.9% and 74.9% of patients in the standard and experimental arms, respectively. Five-year DFS was similar in the two groups (80.8% with preoperative FOLFOX vs. 78.6% with preoperative CRT). Overall survival (hazard ratio for death, 1.04; 95% CI, 0.74 to 1.44) and local recurrence (hazard ratio, 1.18; 95% CI, 0.44 to 3.16) were also similar. Most patients in the response-guided group (90.9%) did not receive CRT and were presumably spared late radiation-associated toxicities. Nevertheless, there was noted to be a higher incidence of severe (grade ≥ 3) adverse events in the FOLFOX group than in the CRT group (41.0% vs. 22.8%). In particular, during neoadjuvant treatment, patients in the FOLFOX group had a higher burden of symptoms such as fatigue, anxiety, nausea and neuropathy. After a year or more following surgery, however, rates of severe symptoms were low in both groups and there were no discernible differences in overall health-related quality of life [56].

Further data to support RT omission comes from the phase III CONVERT trial, which randomized patients with cT2N+ or cT3-4aN0-2 disease with uninvolved MRF to neoadjuvant chemotherapy (four cycles of CAPOX followed by surgery and adjuvant chemotherapy) or neoadjuvant CRT followed by surgery and adjuvant chemotherapy [57]. Patients who received neoadjuvant chemotherapy displayed similar rates of pCR (11.0% vs. 13.8%; *p* = 0.33), downstaging (40.8% vs. 45.6%; *p* = 0.27) and R0 resection (99.6% vs. 99.6%, *p* > 0.99) compared to those who received conventional CRT. There were also reduced rates of perioperative metastases (0.7% vs. 3.1%, *p* = 0.03), diverting ileostomy (52.2% vs. 63.6%, *p* = 0.008) and postoperative complications (18.8% vs. 25.7%; *p* = 0.05). The trial did not meet the primary endpoint of 3-year locoregional recurrence-free survival noninferiority, which was attributed to a lower event rate in both arms leading to wide confidence intervals that exceeded the noninferiority margin. Despite this, 3-year DFS (89.2% vs. 87.9%) and locoregional recurrence-free survival (97.4% vs. 96.3%) were numerically similar in both groups, though there was a trend for worse locoregional recurrence-free survival among patients with tumours < 5 cm from the anal verge treated with neoadjuvant chemotherapy (HR 3.6; *p* = 0.063) [58].

**Table 1:** Total neoadjuvant therapy and radiation omission trials.

				Tumour Factors (%)						Neoadjuvant Toxicity (%)	Organ Preservation (%)					Oncologic Endpoints (%)				
Study	Eligibility	Treatment Arms	n	cT4	cN+	EMVI	MRF	LPLN	≤5 cm from AV	Grade ≥ 3	cCR	ncCR	NOM	TME-Free	pCR	Follow-Up (y)	LRR	DM	DFS	OS
PRODIGE 23 [14,16]	cT3 (at risk for LRR and MCC recommends CRT) or cT4	CRT-TME-mFOLFOX6 x12/cape x8	230	16	90	NA	23	10	36	36	NA	NA	NA	NA	12	7	8	28	63	76
		FOLFIRINOX x6-CRT-TME-mFOLFOX6 x6/cape x4	231	18	90		21	10	38	48					28		5	21	68	82
RAPIDO [15,19]	High risk (at least one of cT4, cN2, EMVI, MRF, LPLN)	CRT-TME-+/-CAPOX x8/FOLFOX4 x12	450	30	92	28	60	15	26	25	NA	NA	NA	NA	14	5	6	30	DrTF: 34	80
		SCRT-CAPOX x6/FOLFOX4 x9-TME	462	32	91	32	62	14	22	48					28		10	23	DrTF: 28	82
STELLAR [21]	cT3-4 or N+	CRT-TME/NOM-CAPOX x6	297	13	84	42	56	NA	49.8	13	4	NA	3	NA	12	3	8	DMFS 75	62	75
		SCRT-CAPOX x4-TME/NOM-CAPOX x2	302	16	86	53	56		48.7	27	11		9		17		11	DMFS 77	55	87
CAO/ARO/AIO-12 [23,24]	Low cT3, mid >cT3b, cT4, cN+	FOLFOX x3-CRT-TME	156	12	86	NA	31	NA	41	CRT 37, CT 22	NA	NA	NA	NA	17	3	6	18	73	92
		CRT-FOLFOX x3-TME	150	18	90		22		41	CRT 27, CT 22					25		5	16	73	92
OPRA [34]	cT3-4N0 or cN+	FOLFOX x8/CAPOX x5-CRT-TME/NOM	158	15	70	20	33	18	NA	41	38	41	71	5y 39	NA	5	LRRFS 94	DMFS 80	71	88
		CRT-FOLFOX x8/CAPOX x5-TME/NOM	166	11	72					34	44	34	76	5y 54	NA		LRRFS 90	DMFS 78	69	85
PROSPECT [55]	cT2N1 or cT3N0-1, sphincter-sparing, MRF ≥3 mm	CRT-TME+/-FOLFOX x8	543	0	63	NA	0	NA	17	23	NA	NA	NA	NA	24	5	2	NA	79	90
		FOLFOX x6+/-CRT-TME+/-FOLFOX x6	585	0	60		0		14	41					22		2		81	90
CONVERT [57,58]	cT2N+ or cT3-4aNany, MRF−	CRT-TME-CAPOX x6	289	26	73	22	0	13	41	8	NA	NA	2	NA	14	3	LRRFS 97	NA	88	94
		CAPOX x4+/-CRT-TME-CAPOX x4	300	28	69	17	0	9	41	12			1		11		LRRFS 96		89	95

cCR, complete clinical response; CRT, chemoradiation therapy; CT, chemotherapy; DFS, disease-free survival; DM, distant metastasis; DrTF, disease-related treatment failure; EMVI, extramural venous invasion; LPLN, lateral pelvic lymph node; LRR, locoregional recurrence; LRRFS, locoregional recurrence-free survival; MCC, multidisciplinary cancer conference; MFS, metastasis-free survival; MRF, mesorectal fascia involvement; ncCR, near-complete clinical response; NA, not available; NOM, non-operative management; OS, overall survival; pCR, pathologic complete response; SCRT, short-course radiation therapy; TME, total mesorectal excision.

Overall, the increasing number of treatment options for stage I–III rectal cancer enables customized treatment that is based on tumour-specific features. This has led to heterogeneity in recommended management of patients with similar radiographic disease burden and risk profile. This heterogeneity was apparent even within our own institution, leading to inconsistencies in clinical recommendations and at times suboptimal setting of patient expectations. The purpose of this initiative was to synthesize the available evidence regarding treatment options and their outcomes in order to reach a consensus-based algorithm that our multidisciplinary gastrointestinal site group can consistently apply to the real-world treatment of patients with stage I–III rectal cancer.

## 3. Development of a Consensus-Driven Evidence-Based Algorithm for the Practical Management of Stage I–III pMMR Rectal Cancer

For the purposes of developing an agreed-upon treatment algorithm that would be readily and consistently applicable in the clinical setting at our institution, we employed a modified Delphi consensus methodology in combination with other qualitative techniques to refine our multidisciplinary group interpretation of the relevant available evidence. The process was designed to enable maximum participation, with the aim of ensuring that all perspectives were voiced and considered. This intention was facilitated by adopting a longitudinal, iterative approach that allowed for reflection, discussion and consultation with external sources of evidence and opinion.

**Figure 1:**
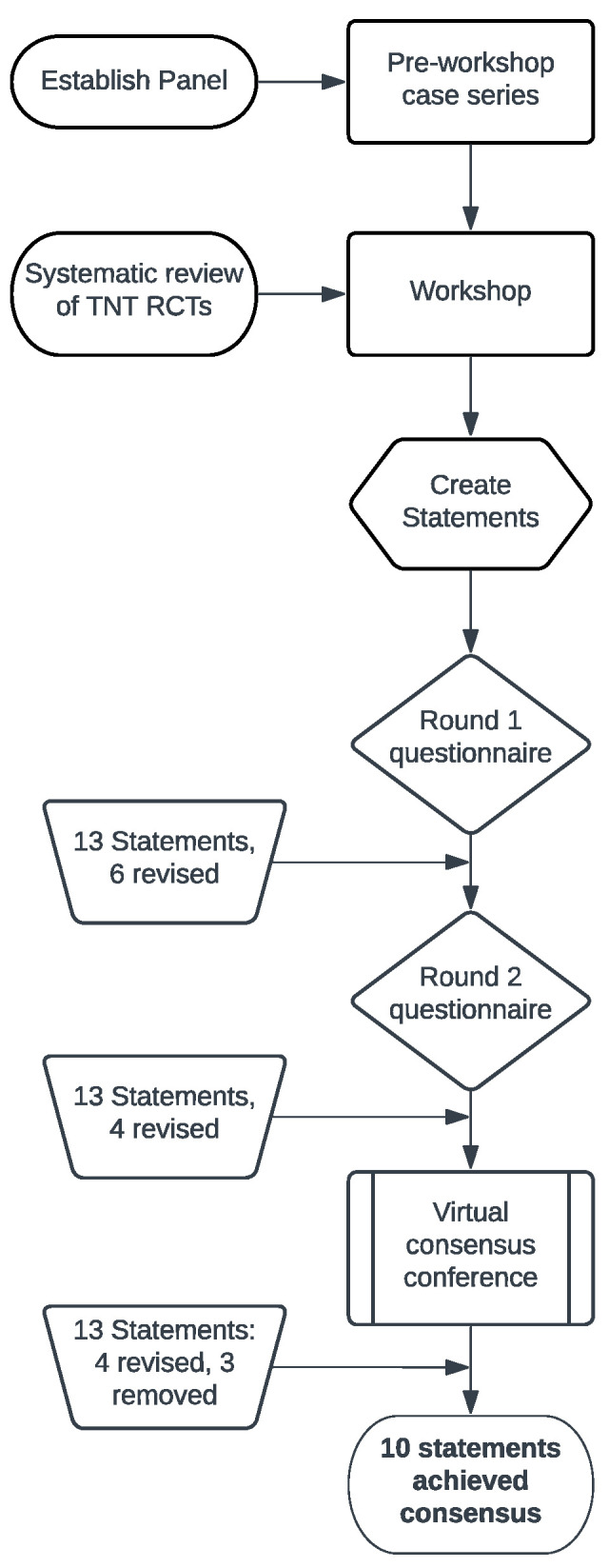
Process used to select and achieve consensus for statements about TNT in stage I–III rectal cancer.

A multidisciplinary group of surgical oncologists (five), radiation oncologists (three) and medical oncologists (four) with expertise in rectal cancer from the Princess Margaret Cancer Centre, Toronto, Canada were approached for consensus review. All surgical oncologists/colorectal surgeons (5/5), three of four radiation oncologists and four of four medical oncologists were able to participate (Figure 1). A pre-meeting case series was prepared including clinical vignettes, MRI synoptic reports and MRI images from seven representative cases identified from our lower GI multidisciplinary cancer conferences to establish topics for the meeting and gain an understanding of the heterogeneity of treatment approaches within the group. For each case, experts were asked about treatment recommendations, the sequencing of treatment modalities such as with TNT, and rationale. Free text comments were also solicited. Participants met virtually for case presentations. Four of seven cases showed agreement (>80%) on applicability of TNT, with significant variability in sequencing of treatments.

An in-person workshop was held in June 2022 to review published evidence, define the methodology for this consensus project and identify topic areas for consensus. In addition, the pre-meeting cases without consensus were reviewed at this meeting to identify areas of discordance and isolate themes. Meeting discussion points were transcribed. Four topic areas for consensus related to TNT terminology/definitions, patient selection, sequencing of treatments and NOM pathways were identified.

**Figure 2:**
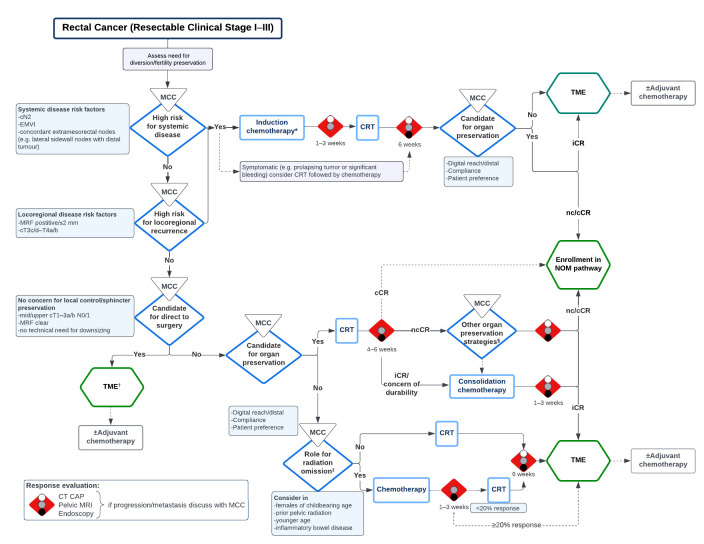
Evidence-based algorithm intended to guide treatment of stage I–III rectal adenocarcinoma. * In select cases, mFOLFIRINOX (PRODIGE-23 protocol) may be recommended by MCC. ^†^ Local excision can be considered in select patients with cT1N0 based on histopathologic risk factors and anatomical considerations. ^‡^ Must meet PROSPECT criteria (cT3N0 or cT2-3N1, MRF ≥ 3 mm, candidate for sphincter-sparing TME). ^§^ Brachytherapy boost and local excision can be considered on a case-by-case basis based on institutional availability and expertise. CRT, chemoradiation therapy; EMVI, extramural venous invasion; iCR, incomplete clinical response; MCC, multidisciplinary cancer conference; MRF, mesorectal fascia; nc/cCR, near-complete/complete clinical response; NOM, non-operative management; TME, total mesorectal excision.

Anonymized Delphi questionnaires were structured as guidance statements/recommendations, and consensus was assessed based on the level of agreement/disagreement for each item. The statements were organized into an electronic questionnaire (Google Forms^®^). Two rounds of voting were sent electronically to 12 participating physicians, with 12 responses being received (100%). For the purpose of this guidance document, consensus was considered achieved when an agreement level of over 80% was reached. Free text comments were elicited during each round of Delphi questionnaires. Two rounds of the electronic anonymous Delphi survey were completed (Figure 1). On the initial round, 13 statements were reviewed by participants and seven reached consensus. Based on free text comments, six statements were revised. For the second round, the statements that did not reach consensus were reviewed and four were revised, while two reached consensus. For the virtual consensus conference, 13 statements were discussed, with revision of terminology for clarity in 4 statements. Three statements were omitted, and ten statements reached consensus (80% agreement) in the following domains: definition, patient selection, sequencing, and NOM/organ preservation. The results of the questionnaires were calculated and reviewed during a virtual consensus group discussion in a non-anonymized fashion with revision of statements. Based on the consensus statements, an algorithm was prepared to provide a practical approach to candidacy and sequencing of treatments. Two additional virtual meetings were held in January 2023, and the algorithm was reviewed and further modified during focus group discussions, with 100% consensus obtained (Figure 2). Most recently, the algorithm and its utilization was updated in May 2025 during the same consensus group’s last meeting. We went on to externally validate the proposed algorithm by arranging virtual multidisciplinary conferences with five high-volume tertiary care cancer centres.

### 3.1. Nomenclature

Initial discussions at the consensus conference focused on the definition of TNT and how to word the recommendations. The TNT nomenclature and sequencing decision nodes were extensively discussed, in particular the use of the term “neoadjuvant” in this setting. Neoadjuvant therapy standardly refers to the administration of therapy before the primary course of treatment, which is usually surgery. In this setting, the group agreed that stage IV (metastatic) disease or borderline resectable/unresectable primary tumours should not be considered for TNT algorithms. Participants all acknowledged that:

• TNT is a management approach for rectal cancer, which intends to deliver anticipated adjuvant therapy, including both systemic chemotherapy and CRT/RT, prior to intended surgery.
• The TNT treatment approach and terminology do not apply to patients who have distant metastatic disease, or whose primary tumour is considered upfront unresectable/borderline resectable.
• Radiographic involved lateral pelvic sidewall nodes (obturator and internal iliac distributions) are considered regional and concordant when associated with a tumour at or below the peritoneal reflection.
• All patients with a new diagnosis of rectal cancer should be discussed at multidisciplinary cancer conference (MCC) prior to a treatment decision being undertaken.

A summary of the consensus statements is presented in Table A1.

### 3.2. Prioritization of Decision Nodes Within Treatment Algorithm

1. In select clinical scenarios, management of presenting symptoms should be prioritized. These include the following:
  - For an obstructing tumour, consider diversion;
  - For significant bleeding or a prolapsing tumour, consider starting with CRT followed by consolidation chemotherapy;
  - Fertility and ovarian functional preservation should be considered in select patients with oocyte retrieval, sperm banking and ovarian transposition.
2. Multiple definitive mesorectal nodes (cN2), concordant lateral pelvic lymph nodes, and EMVI are high-risk radiographic features for development of systemic disease, and should be prioritized as an earlier decision node in rectal cancer treatment algorithms [46,59,60,61,62,63,64,65,66,67]. In these high-risk patients, TNT, rather than preoperative CRT/RT and postoperative chemotherapy, is recommended. The agreed upon sequencing recommendation was to follow an induction chemotherapy followed by CRT pathway. Triplet chemotherapy with mFOLFIRINOX can be considered in select cases for fit patients with a high risk for systemic disease. While the use of this regimen is supported by PRODIGE-23 which showed an overall survival benefit compared to neoadjuvant CRT, the benefit of triplet over doublet chemotherapy remains unknown. The ongoing Janus Rectal Cancer Trial is evaluating the effect of consolidative mFOLFIRINOX versus mFOLFOX/CAPOX on cCR rate and DFS [68].
3. Features suggestive of an increased risk of locoregional recurrence include cT3c/d, cT4, and involved or close (≤2 mm) MRF, and TNT should be considered in this setting with an induction chemotherapy approach [60,61,65,69,70,71]. While the OPRA and CAO/ARO/AIO-12 trials showed that upfront CRT followed by consolidation chemotherapy and the associated longer interval to restaging/surgery resulted in sustained cCR/organ preservation or better pCR without adverse effect on DFS and distant metastases-free survival, induction chemotherapy followed by CRT was recommended as these tumours are less likely to achieve a cCR regardless of the sequencing of TNT treatment, and the higher pCR rate (CAO/ARO/AIO-12) did not result in improved survival or lower local recurrence rates [24,33,72,73,74]. This approach can also mitigate potential operative risks and morbidity associated with increased radiation-to-surgery intervals [75,76,77].
4. In lower-risk tumours, upfront TME surgery can be considered. These include mid- or upper-rectal cT1N0 not amenable to local excision and cT2-3abN0/1 tumours with clear MRF, no concern for local control or sphincter preservation, and no technical need for downsizing. Conversely, TNT can be considered for lower-risk mid- and upper-rectal tumours (cT3N0) to facilitate maximal downsizing in the case of perceived challenging resection (i.e., bulky and anterior).
5. Distal rectal tumours that would otherwise be considered for an abdominoperineal resection or coloanal anastomosis may be candidates for a NOM/organ preservation approach. Given the implications on permanence of stoma and bowel function, it is preferred if the tumour is within digital reach, though NOM can be considered on a case-by-case basis for tumours beyond digital reach based on patient factors (e.g., morbid obesity). In select patients, this may come as a consequence of completion of treatment with endoscopic, radiographic and clinical findings of a complete response. In other patients, treatment selection and sequencing will be carried out with the intent of inducing a cCR and therefore a non-operative approach to management. In both situations, the decision should be made by the multidisciplinary team in the context of an extensive patient discussion.
6. CRT with optional consolidation chemotherapy can be considered in the setting of cT1N0 distal (<5 cm from the anal verge) tumours that are not amenable to local excision (e.g., high-risk histopathologic features or tumour anatomical constraints) or cT2-3bN0 distal tumours that would require an abdominoperineal resection or a coloanal anastomosis with the goal of organ preservation. Considerations for consolidation chemotherapy after CRT in the setting of a cCR should be based on multidisciplinary discussion regarding the benefits balanced against the risk of over-treatment. Data supporting organ preservation strategies integrating a combination of neoadjuvant CRT, chemotherapy, brachytherapy boost and/or transanal local excision are emerging [78,79,80,81]. Based on institutional availability and expertise, these approaches may be considered following MCC discussion on a case-by-case basis in patients who sustain a near-cCR, but this falls beyond the scope of this article.
7. Systemic therapy and restaging can be performed to determine selective RT utilization prior to TME surgery in patients with tumours where there is no concern for local control or candidacy for NOM, and existing rationale for omission of RT, such as in a female of childbearing age, younger patients, patients with inflammatory bowel disease, or a patient who has undergone prior pelvic radiation, systemic therapy and restaging can be performed to determine selective radiation utilization prior to TME surgery. The role of upfront systemic chemotherapy to identify patients who may derive minimal benefit from preoperative RT and for whom it might be safely avoided was discussed and is the rationale for the PROSPECT and CONVERT randomized trials.
8. Patients considered for NOM pathways should be enrolled in a comprehensive and close active surveillance pathway by an experienced high-volume centre. Surveillance should be multimodal, including DRE, endoscopic surveillance, biochemical testing, and radiographic exams (MRI and CT). Frequency of surveillance will vary by institution but should follow published recommendations (i.e., NCCN).
9. For patients with non-metastatic rectal cancer, the decision to adopt short-course RT remains institution-dependent. To maximize the likelihood of NOM or downsizing of a bulky primary, our preference is to pursue long-course CRT. Moreover, we would caution utilization of short-course RT in patients with T4 tumours or those with threatened/involved MRF given increased rates of locoregional failure (12% vs. 8%; *p* = 0.07) and locoregional recurrence (10% vs. 6%; *p* = 0.027) in the RAPIDO trial, particularly with involvement of the MRF [19].

## 4. Summary and Future Directions

The impetus for this algorithm was the rising utilization of TNT for rectal adenocarcinoma in the context of significant heterogeneity in approaches and indications. Given the potential for over-treatment with the incorporation of TNT into rectal cancer treatment algorithms, a targeted and thoughtful approach to selecting patients is required. In rectal adenocarcinoma, tailoring of treatment intensity has the potential to promote NOM or organ preservation strategies, reduce recurrence risk and minimize toxicities. An inclusive, pragmatic, evidence-based algorithm is presented here.

As management strategies evolve, alternative treatment strategies and biomarkers for recurrence or minimal residual disease will need to be incorporated into treatment paradigms. While radiation intensification with contact brachytherapy and omission strategies are now supported by randomized trial data, approaches that intensify chemotherapy with triplet regimens or de-intensification strategies with oxaliplatin omission will be informed by trials such Janus and CCHOWW, respectively [68,82]. Furthermore, incorporation of transanal local excision following neoadjuvant radiation or chemotherapy is an emerging strategy to extend organ preservation that will be further refined by the ongoing NEO-RT trial [83].

There is emerging evidence to support the use of response biomarkers, such as ctDNA, to help predict post-treatment outcomes and determine whether additional treatment should be considered in a response-adaptive approach or to tailor surveillance regimes. Studies of localized colorectal cancer have consistently shown that ctDNA testing reliably detects minimal residual disease after curative-intent surgery and predicts recurrence with good sensitivity and extremely high specificity [84,85,86,87,88]. Specifically in rectal cancer, the GALAXY study demonstrated a DFS benefit with adjuvant chemotherapy among ctDNA-positive patients following upfront TME, whereas there was no benefit among ctDNA-negative patients [89]. In the setting of neoadjuvant chemotherapy, ctDNA dynamics provide an early response evaluation as early as after two cycles of chemotherapy that predicts poor response, which could potentially lead to adaptive treatment sequencing [90]. Randomized control trials are needed to validate ctDNA-guided strategies in rectal adenocarcinoma.

## Data Availability

No new data were created or analyzed in this study.

## Abbreviations

The following abbreviations are used in this manuscript:

cCR	Clinical complete response
CRT	Chemoradiation therapy
DFS	Disease-free survival
dMMR	Mismatch repair deficient
DRE	Digital rectal examination
EMVI	Extramural venous invasion
MCC	Multidisciplinary cancer conference
MRF	Mesorectal fascia
NOM	Non-operative management
pCR	Pathologic complete response
pMMR	Mismatch repair proficient
RT	Radiotherapy
TME	Total mesorectal excision
TNT	Total neoadjuvant therapy

## Appendix A

**Table A1:** Consensus statements.

Statement	Consensus (≥80%) Reached (% Agreement)
Patient selection	
TNT, rather than preoperative CRT and postoperative chemotherapy (i.e., in situations where systemic therapy is indicated), is recommended in the following settings: Positive mesorectal nodes; Concordant extramesorectal lymph nodes (i.e., regional); EMVI.	92.3%
TNT can be considered in the following settings: When there is significant concern for R1 resection (cT4, bulky cT2/T3, involved or close MRF margin (≤2 mm)); For lower-risk distal tumours (cT2/cT3N0) that would require either an abdominoperineal resection (APR) or a coloanal anastomosis, with the goal of non-operative management/organ preservation; For lower-risk mid- and upper-rectal tumours (cT3N0) to facilitate maximal downsizing in the case of perceived challenging resection (i.e., bulky and anterior).	100%
TNT is not recommended in the following settings: Mid/upper-rectal cT1/cT2N0; Upper rectal cT3N0 with clear MRF margin; Distal cT1N0 tumours ^a^.	92.3%
Sequencing	
Induction chemotherapy (chemotherapy before CRT) is recommended in tumours with high-risk features of development of systemic disease including: EMVI; definitive mesorectal lymph nodes; Concordant lateral pelvic sidewall nodes (i.e., regional).	100%
When considering TNT for lower-risk distal tumours (cT2N0/cT3N0) that would require either an APR or a coloanal anastomosis, CRT with interval reassessment to determine the role of consolidation chemotherapy is recommended.	84.6%
Where maximal downsizing is desired (e.g., T4, involving/close to sphincter, bulky, mucinous, or threatened MRF (≤2 mm)) or in patients who are highly symptomatic on presentation, optimal sequencing of treatment modalities should be discussed on a case-by-case basis.	84.6%
Non-operative management/organ preservation	
Following completion of intended TNT, patients with a complete clinical response (i.e., no evidence of residual tumour on digital rectal examination, rectal MRI, and direct endoscopic evaluation) can be offered a watch-and-wait/active surveillance approach by an experienced multidisciplinary team.	84.6%

^a^ If local excision is not indicated and considering non-operative management, CRT followed by reassessment with consideration of systemic therapy for residual disease is recommended.
